# Percutaneous Septal Ablation in Hypertrophic Obstructive Cardiomyopathy: From Experiment to Standard of Care

**DOI:** 10.1155/2014/464851

**Published:** 2014-05-06

**Authors:** Lothar Faber

**Affiliations:** Department of Cardiology, Heart and Diabetes Center North Rhine-Westphalia, University Hospital of the Ruhr University Bochum, Georgstraße 11, 32545 Bad Oeynhausen, Germany

## Abstract

Hypertrophic cardiomyopathy (HCM) is one of the more common hereditary cardiac conditions. According to presence or absence of outflow obstruction at rest or with provocation, a more common (about 60–70%) obstructive type of the disease (HOCM) has to be distinguished from the less common (30–40%) nonobstructive phenotype (HNCM). Symptoms include exercise limitation due to dyspnea, angina pectoris, palpitations, or dizziness; occasionally syncope or sudden cardiac death occurs. Correct diagnosis and risk stratification with respect to prophylactic ICD implantation are essential in HCM patient management. Drug therapy in symptomatic patients can be characterized as treatment of heart failure with preserved ejection fraction (HFpEF) in HNCM, while symptoms and the obstructive gradient in HOCM can be addressed with beta-blockers, disopyramide, or verapamil. After a short overview on etiology, natural history, and diagnostics in hypertrophic cardiomyopathy, this paper reviews the current treatment options for HOCM with a special focus on percutaneous septal ablation. Literature data and the own series of about 600 cases are discussed, suggesting a largely comparable outcome with respect to procedural mortality, clinical efficacy, and long-term outcome.

## 1. Etiology, Pathogenesis, and Pathophysiology of HCM

Hypertrophic cardiomyopathy (HCM [1–70]) is a cardiac condition morphologically characterized by unexplained myocardial hypertrophy. Extent and distribution of wall thickening are highly variable; the interventricular septum is most often involved, while the right ventricle is rarely affected. The prevalence of the disease is considered to be around 0.2%; in >50% of patients HCM has a familiar background [3, 6–8]. Inheritance shows an autosomal-dominant pattern, with an incomplete and highly variable penetrance. Mutations have been found in >2 dozens of genes coding for sarcomeric proteins or those involved in myocardial energy metabolism; the condition therefore has been characterized as a “sarcomeric disease” [42–48]. Histologically, the prominent findings in HCM are myocardial disarray, hypertrophy, and fibrosis [49–59]. Not only the myocardial walls but also the coronary vasculature walls are often thickened which may decrease coronary reserve and lead to myocardial ischemia in the absence of occlusive atherosclerosis. In addition, myocardial bridging is a rather frequent finding, and mitral valve leaflets may be elongated [13–15].

Left ventricular systolic function as expressed by the ejection fraction is normal in the vast majority of patients, although modern imaging techniques frequently show impaired longitudinal systolic deformation of the affected myocardium. In addition, fibrosis and hypertrophy lead to increased myocardial stiffness and impairment of diastolic left ventricular function early in the disease process [5, 6, 8, 31, 32, 54–57]. Elevated filling pressures and a reduced stroke volume with stress may thus be present as in other entities characterized as “heart failure with preserved ejection fraction” (HfpEF), and left atrial dilatation is a typical morphological finding in HCM patients. A late stage of the disease with a dilated left ventricle and reduced ejection fraction may be observed in up to 5% of cases.

Independent from the functional limitation, a wide spectrum of supraventricular and ventricular arrhythmias may occur at every stage during the disease course. Again, fibrosis and disarray play an important role as the arrhythmogenic substrate; myocardial ischemia due to hypertrophy and thickened vessel walls may be an additional trigger [6, 8, 51, 58, 59]. Sudden cardiac death is a feared complication of the disease and sometimes its first manifestation. Among young (<35 years) athletes dying suddenly, HCM (usually the nonobstructive phenotype) is considered to be responsible for about 30%. The dissociation between morphology, functional status, and arrhythmogenic risk is a major problem of HCM management. Sudden cardiac death, often occurring during or after strenuous exercise, is more common in younger and previously asymptomatic patients. Stroke and heart failure related death seems to prevail in elderly cohorts.

**Figure 1:**
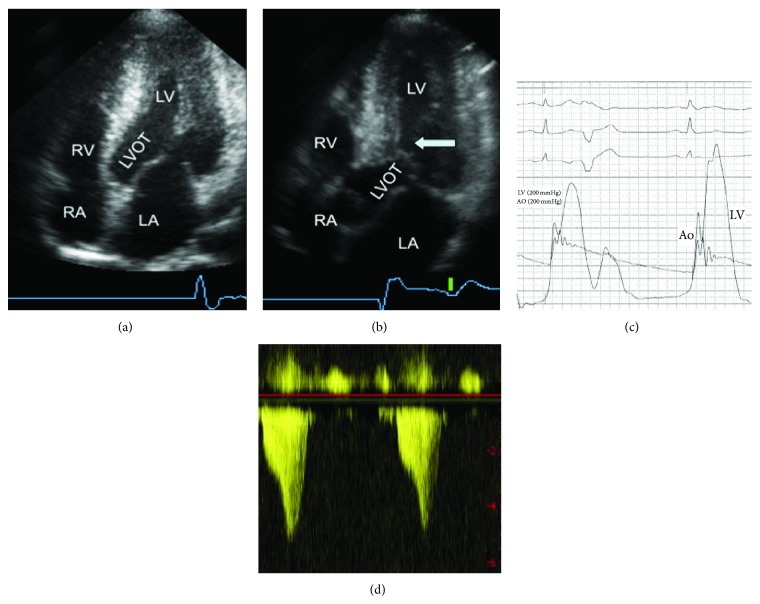
2D-echocardiographic findings in hypertrophic nonobstructed cardiomyopathy (HNCM, (a)) with predominant thickening of the apical segments and a wide open, unobstructed outflow tract (LVOT) and in hypertrophic obstructive cardiomyopathy (HOCM, (b)) with a protruding subaortic septum making systolic contact with the mitral valve (SAM-phenomenon, arrow). (c) shows simultaneous pressure tracing from the LV and the aorta demonstrating the outflow gradient and the Brockenbrough sign. The corresponding Doppler profiles are shown in (d). The gradient increases from 40 to 140 mm Hg. The typical CW-Doppler flow profile of left ventricular outflow obstruction in HOCM has a late-peaking signal indicating dynamic obstruction involving contracting muscle as opposed to the more symmetrical signal of fixed valvular stenosis. The peak pressure gradient equals 4  × (peak velocity)^2^. LA: left atrium; RA: right atrium; LV: left ventricle; Ao: aorta; and IVS: interventricular septum.

**Figure 2:**
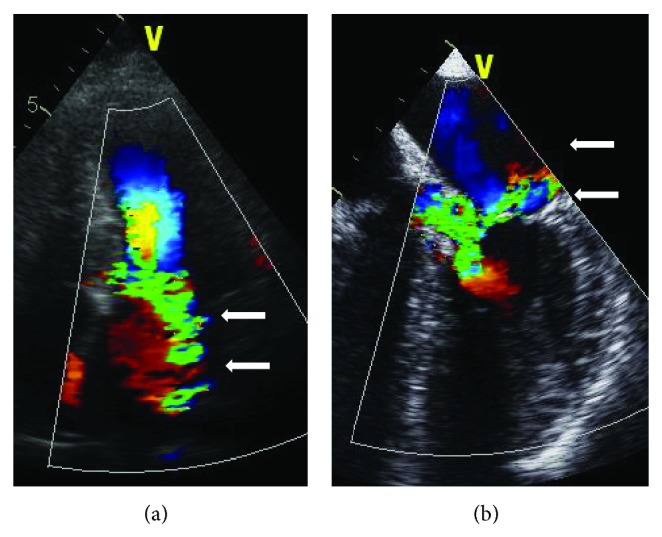
Typical mitral regurgitation associated with SAM and subaortic LVOT obstruction with a posterolateral jet orientation (arrows) in a transthoracic (a) and transesophageal view (b). LA: left atrium; RA: right atrium; LV: left ventricle; Ao: aorta; and IVS: interventricular septum.

An important distinction in HCM is between the nonobstructive (hypertrophic nonobstructive cardiomyopathy: HNCM) and the obstructive (HOCM) phenotype of the disease (Figure 1). Dependent on the distribution of hypertrophy within the left ventricle, the septal curvature, the size and configuration of the mitral valve, and left ventricular loading conditions, about 60–70% of HCM patients develop a dynamic obstruction between a “high-pressure” and a “low-pressure” compartment of the left ventricle [2–6, 8, 9, 19, 33–35]. Typically this obstruction is located between the subaortic septum and parts of the mitral valve (“SAM” phenomenon: systolic anterior movement) and is associated with mitral regurgitation. SAM-associated mitral regurgitation shows a typical posterolateral jet direction that can be used for differentiation towards primary mitral regurgitation (Figure 2). In a minority of cases outflow obstruction may be located in the midcavity region, in the apex, or occasionally in the right ventricular outflow tract. The hemodynamic significance of obstruction seems to depend on the size of the LV compartment that is working against increased afterload; apical gradients are considered to be less significant. A substantial degree of variability has been described regarding gradient severity, and provocation (by physical exercise, preload reduction, inotropic agents, or postextrasystolic augmentation) is essential to distinguish between HNCM and HOCM both during echocardiographic and invasive hemodynamic studies [6, 8, 19].

## 2. Symptoms, Clinical Workup, and Natural History in HCM

Typical symptoms in HCM patients are dyspnea, angina, or dizziness on exertion. A marked day-to-day variability is typical for the disease. Palpitations or syncope occurring both with and without exercise are reported by 20–30% of patients. Recurrent syncope and a family history of sudden cardiac death (at <45 years) have to be actively asked for because these features are considered risk factors [20] for sudden arrhythmogenic death. On the other hand, a severe HCM phenotype on imaging studies does not necessarily preclude normal exercise capacity or even athletic performance.

Cardiac auscultation is usually normal in patients with HNCM. The characteristic auscultatory finding in HOCM is the variable systolic murmur which accentuates with preload reduction (e.g., with a Valsalva maneuver) and which diminishes with increase of afterload (e.g., with squatting). All types of ECG changes may be present; the typical ECG changes in HNCM are “giant negative T waves” and “pseudoinfarction Q waves” in HOCM. ECG changes may precede the phenotype on imaging studies by decades. Holter monitoring should be performed for risk stratification in every HCM patient since the finding of nonsustained VT's is another risk marker. Stress testing is useful to objectively measure the degree of functional limitation and to check the blood pressure response to exercise which is considered another risk factor for sudden cardiac death (see below).

The diagnosis of HCM can usually be made by noninvasive imaging techniques (echocardiography with its different modalities, cardiac magnetic resonance imaging, and multislice computed tomography). A multimodal approach is useful in many patients since the full extent of wall thickening is sometimes missed by 2-dimensional echocardiography, and cardiac magnetic resonance imaging with contrast enhancement allows for additional assessment of fibrosis. The degree and the distribution of hypertrophy are highly variable, ranging from isolated thickening of individual myocardial segments that merely exceed the normal LV wall thickness of <12 mm up to diffuse and massive hypertrophy of >50 mm. A wall thickness of >30 mm has to be actively looked for since this is the fifth risk factor for sudden cardiac death [6, 8, 17, 20].

Invasive studies are needed to exclude coexistent coronary artery disease, to visualize the anatomy of the septal perforator arteries if septal ablation is considered, and to perform endomyocardial biopsy if a myocardial storage disease is suspected. The level of suspicion for such a storage disease should be high in presence of a low-voltage ECG. A prevalence of storage diseases of up to 10% has been reported in “HCM” series [6, 8]. Diastolic LV performance and the outflow gradients can also be assessed invasively. The role of invasive electrophysiology studies for risk stratification is uncertain.

Natural history in HCM is highly variable [5, 6, 8, 28, 37–41]. In most cases the diagnosis is made during adolescence until early adulthood, and symptoms are slowly progressive. Disease manifestation in childhood is considered prognostically ominous. Late manifestation, however, is typical in carriers of the myosin-binding protein C mutation. Prognosis is determined by arrhythmic events in younger patients, typically independent of symptoms in this group, and by cardiac failure and stroke in elderly patients. In nonselected cohorts, the annual mortality rate is reported to be around 1%/year; in high-risk group this figure rises up to 5-6% [6, 8, 19].

## 3. Therapeutic Considerations for HOCM: Risk Management, Medical Therapy, Pacemakers, and Surgery

Whether or not obstruction or symptoms are present, HCM patients should not engage in competitive sports [6, 8, 71]. A limitation with respect to moderate physical activities in asymptomatic patients, however, does not seem to be justified. Outflow obstruction may exacerbate with alcohol intake, and the turbulent flow in the LVOT together with the obstruction-associated mitral regurgitation includes an increased risk for infective endocarditis [72, 73]. HCM patients in atrial fibrillation are endangered by thromboembolic stroke; oral anticoagulation is thus mandatory in these cases [6, 8]. All HCM patients should be risk-stratified [6, 8, 20, 74–82] since the implantation of an ICD reliably reduces arrhythmogenic cardiac events.

Risk stratification in HCM is based on the presence versus absence of five major risk factors, each one with a relatively low positive individual predictive value. However, combining them their significance considerably increases. These risk markers are (see above) as follows:

- a “malignant” family history (of sudden cardiac death at <45 years),
- recurrent unexplained syncope,
- nonsustained ventricular tachycardia on Holter monitoring,
- inadequate blood pressure rise with exercise (i.e., failure to rise by >20 mm Hg or a fall of >20 mm Hg after an initial rise),
- excessive LVH (>30 mm) in any region.

Patients without any of the listed risk markers seem to have a favorable prognosis, although cases of SCD have been reported with a completely negative list of risk factors. Other aspects that suggest a benign disease course are a normal or near-normal ECG, advanced age >65 years at diagnosis, and a preserved exercise tolerance on cardiopulmonary stress testing. On the other hand, in individuals carrying two or more of these risk markers, ICD implantation should be strongly considered. Whether just one risk factor is sufficient for primary ICD prophylaxis is controversially discussed. In our practice a malignant family history is a strong argument for an ICD even if this is the only risk marker.

Recently, the documentation of areas of marked late gadolinium enhancement/fibrosis on cardiac MRI has been linked to an increased risk [8, 21–23, 53, 57, 67]. In addition, very early onset of the disease, the presence of an apical aneurysm and of myocardial bridging, objective signs of myocardial ischemia, marked left atrial dilatation, and supraventricular tachyarrhythmias have been linked to future adverse events, although in smaller patient cohorts. Patients with a late dilated stage of the disease seem to be a high-risk category of its own with a very unfavorable prognosis.

Medical therapy with negatively inotropic drugs (beta-blockers, calcium antagonists of the verapamil type, and disopyramide) is the first line of treatment in order to reduce symptoms and improve quality of life [83–86] in patients with HOCM. Additional antifibrillatory effects may be present for beta-blockers, while verapamil is supposed to have a positive effect on diastolic LV function. Beta-blocker dosage for symptom control should be uptitrated to a resting heart rate of 50–60 beats/min. The effect of disopyramide on obstruction seems to exceed that of the two other drugs; however, disopyramide is no longer available in central Europe.

The antiarrhythmic properties of the different drugs are welcomed in many patients. On the other hand, latent conduction abnormalities may exacerbate in individual cases. Furthermore, about 5–10% of patients may have a paradoxical hemodynamic response to verapamil. The initiation of treatment with verapamil and disopyramide therefore should be monitored closely. Overall, in many patients, the effect of drug treatment vanishes over the years, and none of these strategies are really “evidence-based.” Drugs that lead to a marked pre- or afterload reduction or those with positive inotropic effects are contraindicated in HOCM since they may produce drastic exacerbation of obstruction and hemodynamic collapse.

Medical therapy in HCM without obstruction, either in “deobstructed” HOCM after a septal reduction intervention or in primary HNCM, may be understood as HFpEF treatment. In order to optimize left ventricular filling time, heart rate should be tightly controlled using beta-blockers or verapamil-type calcium antagonists. Diuretics and ACE inhibitors/AT receptor antagonists may be used for signs of congestion or concomitant hypertension. Occasionally, an outflow tract obstruction may be produced in initially nonobstructive patients by vigorous afterload reduction; thus we again recommend echo-Doppler monitoring of the initial phase of therapy. Animal experiments and a recently published study in human HNCM [86] suggest inhibition or even reversal of fibrosis with AT receptor antagonist treatment.

Atrial fibrillation with loss of active ventricular filling-in is often associated with a considerable drop in exercise tolerance and an increased risk of embolic events. Anticoagulants should be promptly administered, and Amiodarone can prevent recurrence of atrial fibrillation. Ablation therapy of atrial fibrillation is an additional option; however, outcomes are less favorable as compared to patients without structural heart disease. End-stage disease should be treated as severe heart failure of other etiologies, including modern assist device strategies and heart transplantation.

**Figure 3:**
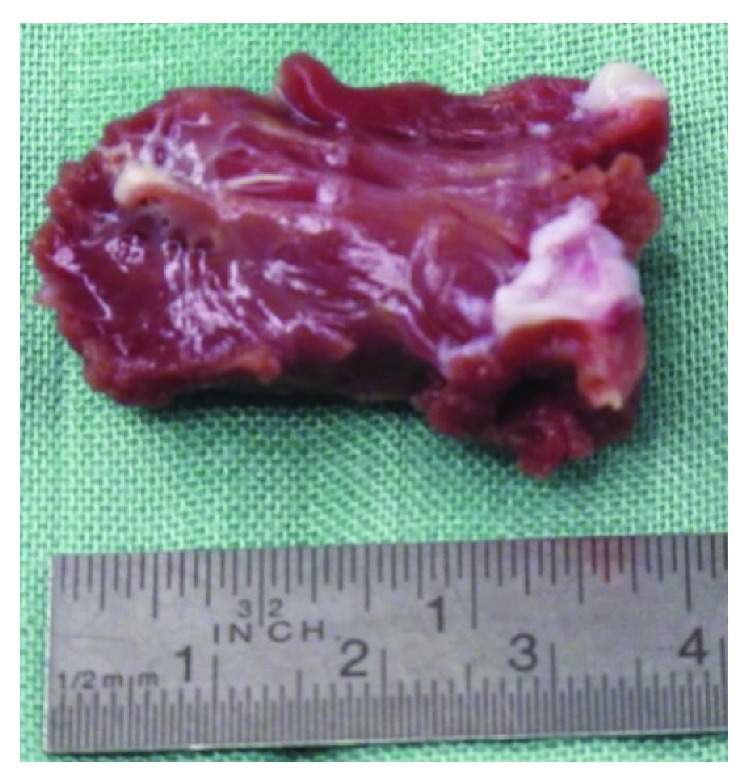
Septal myocardium removed during a myectomy procedure. According to septal thickness and location of the obstruction, a block of myocardium of 4.0 × 1.5 × 0.8 cm was removed from the subaortic septum.

**Figure 4:**
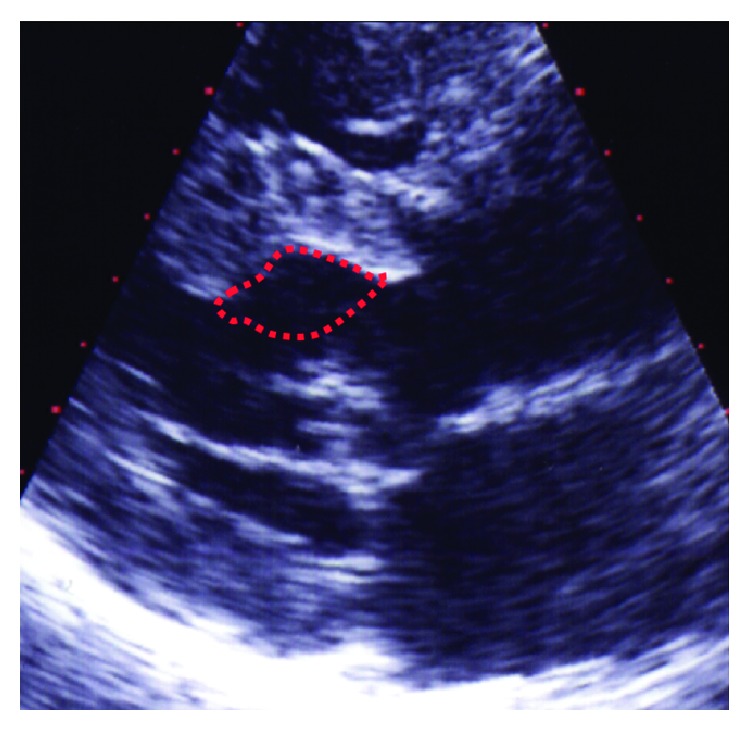
Echocardiographic visualization of the septal trough (dotted line) produced by a myectomy procedure.

Surgical myectomy, developed in the late fifth and the sixth decade of the 20th century, traditionally has been the treatment of choice for HCM patients with drug-refractory symptoms and significant outflow obstruction [87–97]. The procedure aims at removing a part of the protruding septal myocardium (Figure 3) via a transaortic approach and leaves a clearly visible septal trough on imaging studies (Figure 4) and usually a left bundle-branch block on the surface ECG in >50% of the patients treated (Figure 6). The depth and extent of septal resection can be tailored to the individual anatomy, thus also addressing midcavity obstruction or papillary muscle abnormalities if present. Furthermore, valvular correction/replacement or coronary bypass grafting can be combined with the reduction of septal myocardium if necessary. Perioperative monitoring by transesophageal echocardiography has become a routine procedure. The rate of pacemaker dependency is reported to be ≤5%.

Reports on >2000 patients undergoing (isolated) myectomy consistently demonstrated clinical and hemodynamic success rates of >90% together with operative mortality rates that finally were reduced to <1-2% in experienced centers. A favorable effect on the hypertrophic process and a positive prognostic influence [94] are suspected from long-term observations of postmyectomy patients; however, a randomized study against medical treatment does not exist. Favorable results of myectomy were also reported in specific subsets including pediatric patients as well as cases with atypical or midcavity obstruction. Taking all this together, myectomy has set the standard of safety and efficacy of treatment for symptomatic obstructive HCM; and all alternatives should be measured against this standard.

After some observations concerning LVOT gradient reduction with pacing, dual-chamber pacemaker implantation was introduced as a less invasive alternative to myectomy in the ninth decade of the 20th century. Pacing from the RV apex with a short AV-delay may be understood as a combination of a global negative inotropic effect and some outflow tract opening due to delayed activation of the basal septum. A gradient reduction of 50–90% has been reported. Enthusiasm for this approach, however, was tempered since a considerable placebo effect became obvious in several randomized trials [98]. At present, we consider AV sequential pacing a “niche indication” for

1. patients with left bundle-branch block (and thus a very high risk for complete AV block during septal ablation (see below)),
2. patients who need an ICD for risk reduction anyway,
3. selected patients with isolated midcavity obstruction.

## 4. Septal Ablation: From Experiment to Standard of Care

From 1995 onwards, therapeutic options for HOCM dramatically changed by the introduction of percutaneous septal ablation [99–140]. In 1994, after obtaining ethical approval for a limited series of cases to undergo this new procedure, Sigwart performed the first three septal ablations in elderly, highly symptomatic HOCM patients who were unable to tolerate surgical myectomy. The positive results of these first cases were published in 1995 [99], followed by a widespread adoption of the new technique.

**Figure 5:**
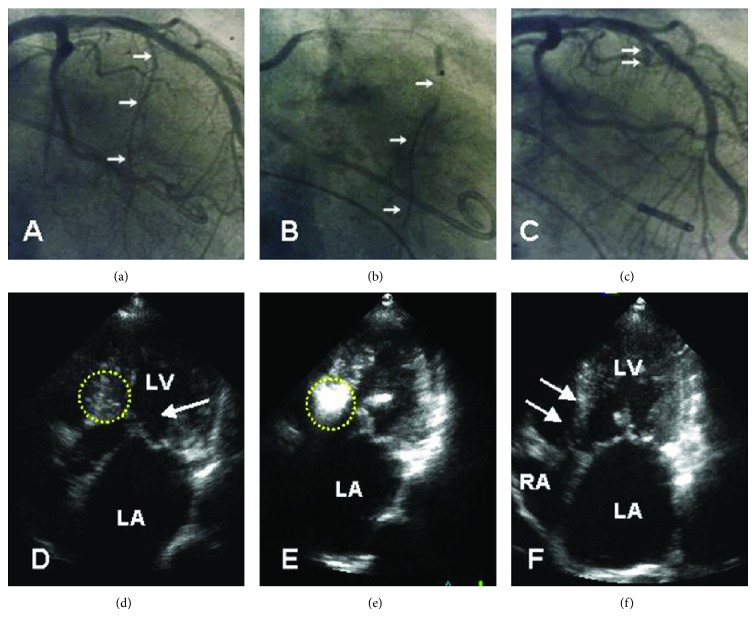
Angiographic ((a)–(c)) and echocardiographic ((d)–(f)) aspect of an echotargeted septal ablation procedure (in our practice denominated as PTSMA). A guidewire is advanced into the target vessel (arrows in (a)). Subsequently, an over-the-wire balloon is introduced. The correct position and fit of the balloon are verified by contrast injection (arrows in (b)) through the central catheter lumen. The vessel stump after alcohol injection and removal of the balloon is shown in (c) (arrows). In (d), the dotted circle marks the septal target area including the SAM-septal contact zone. Contrast injection into the target vessel (e) precisely highlights this area. After 3–6 months, akinesia and thinning of the subaortic septum are clearly visible, comparable to a myectomy trough.

The septal ablation procedure produces a circumscript necrosis by injection of 96% ethanol (or other toxic agents; see below) into a septal perforator artery supplying the septal bulge involved in outflow obstruction (Figure 5). Several components of the procedure had earlier been tested or used clinically and in other scenarios. In the early 1980 years, the group of Sigwart reported on the effect of temporary balloon occlusion within a coronary vessel on myocardial function and thickening [100]. Brugada and coworkers, among others, used the injection of absolute ethanol into coronary arteries to eliminate arrhythmogenic foci [101]. The group of Kuhn and coworkers [102, 103] reported on temporary gradient reduction in HOCM following temporary balloon occlusion of septal perforator arteries. Even the use of intraprocedural contrast echocardiography had been outlined in a research proposal as early as 1989 [104]. Several acronyms have been introduced for the technique (in alphabetical order and probably incomplete): alcohol/ethanol septal ablation (ASA/ESA), nonsurgical myocardial reduction (NSMR), percutaneous transluminal septal myocardial ablation (PTSMA), or transcoronary ablation of septal hypertrophy (TASH), reflecting slightly different procedural strategies and/or operator preference.

**Figure 6:**
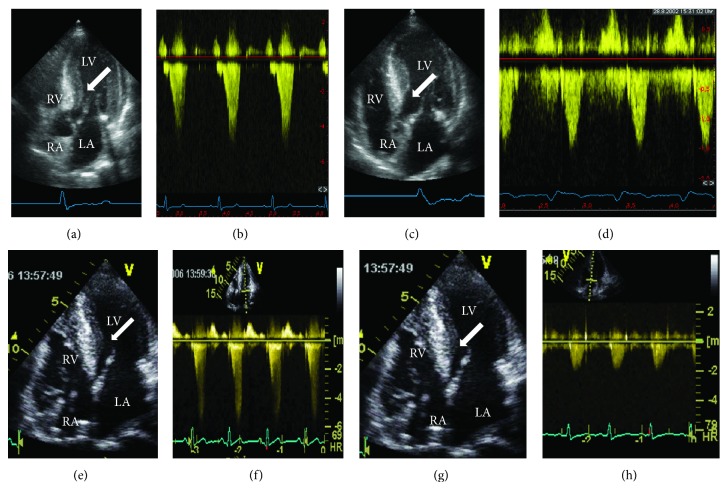
Echocardiographic aspect of HOCM before/after a myectomy ((a)–(d)) and after a percutaneous septal ablation ((e)–(h)). Both cases show marked thickening of the midcavity and subaortic septum (arrows in (a) and (e)) at baseline together with a substantial outflow acceleration to >5 m/s corresponding to an outflow gradient of 100 mm Hg at rest ((b) and (f)). After the respective intervention there is thinning of the subaortic septum (arrows in (c) and (g)) and normalisation of LV outflow to <2 m/s, that is, absence of a resting gradient. The different ECG patterns of QRS widening with a LBBB pattern in C/D after myectomy and a RBBB pattern after septal ablation are also visible. LA: left atrium; RA: right atrium; RV: right ventricle; and LV: left ventricle.

The septal lesion produced by the procedure often closely resembles a myectomy trough (Figures 5 and 6), and it also reproduces the hemodynamic effect of a surgical myectomy with reduction/elimination of the outflow gradient, SAM, and the SAM-associated mitral regurgitation. After the procedure, about 50–60% of the patients show a right bundle-branch block pattern on surface ECG and have transient complete heart block during the procedure. Across all reported series including the learning curve of the individual investigator groups, periprocedural mortality of septal ablation was 1–4%, at present 1-2%. This holds true both for several single-center series and for multicenter registries [115, 116, 127]. The injected ethanol doses gradually decreased over the years (from >5 to 1–3 mL), leading to smaller infarctions and less AV conduction problems. However, the rate of pacemaker implantation still varies considerably (between <5 and up to 20%, in patients with preexisting left bundle-branch block: >60%; see above). Following a local remodeling process, the morphologic and hemodynamic treatment result should be judged no earlier than after 3–6 months. At that time point, gradients usually are reduced by 80–90%, associated with an increase in exercise capacity by 20% and an improvement of diastolic LV function markers. During the past two decades septal ablation has gained wide acceptance as the nonsurgical alternative of choice for patients with hypertrophic cardiomyopathy, significant outflow obstruction, and symptoms refractory to medical treatment.

### 4.1. Septal Ablation Procedure

A detailed description of the technique has been repeatedly published by our and other groups, differing in several technical aspects [99, 106, 108, 109, 112–116]. In general, two phases of technical development can be described. From roughly 1995 to 1998, during a phase of initial deployment of the new technique, relatively high doses (in some cases >10 mL) of ethanol were injected almost always into the first septal perforator artery, not guided by any imaging techniques. This era, including the early learning curve in most groups, resulted in relatively good clinical efficacy, with reductions in gradient and improvement in symptoms, but with rather high complication rates, including complete heart block requiring pacemaker implantation in an unacceptable large proportion of patients undergoing the procedure and also probably underreported, distant myocardial infarction or death from inadvertent spillage of ethanol.

During the next phase, roughly between 1999 and 2009, the incorporation of myocardial contrast echo as a guide in order to select the correct septal perforator substantially increased procedural safety. Furthermore, careful follow-up of postablation patients demonstrated that it was not necessary to completely eliminate obstruction during the ablation session, leading to a substantial reduction of the injected amount of alcohol (currently to roughly 2 mL or 1 mL per cm of myocardial thickness in the target region). The pacemaker implantation rate was brought down by scoring systems to estimate the risk of procedure-related persisting or recurrent conduction problems.

There is still consensus that a temporary pacemaker lead should be routinely inserted in all patients. The outflow gradient may be monitored using simultaneous pressure recordings from the left ventricular apex and the ascending aorta. A standard short (10–12 mm) over-the-wire balloon catheter is introduced into the target septal branch presumed to be responsible for the blood supply to the septal area involved in obstruction. The balloon is inflated, and the effect on obstruction is measured.

In contrast to other techniques that strongly rely on this effect, in our practice as well as in most other centers the correct vessel selection is assured by injecting 1-2 mL of a nontoxic echocardiographic contrast agent through the central lumen of the balloon catheter under simultaneous transthoracic echocardiographic monitoring. This approach exactly shows the septal area that will be attacked, that is, the future area of necrosis (Figure 4). Opacification of any other cardiac structures has to be securely excluded [109]. Currently, in about 15% a target vessel change is necessary based on echocardiographic findings (usually contrast in areas distant from the septal target region) or for the same reason the procedure has to be stopped [120, 130]. Only if the target region is correctly marked, 1–3 mL of 96% alcohol (i.e., 1 mL per 1 cm of septal thickness) is slowly injected through the central lumen of the balloon catheter under analgesic medication (5–10 mg of morphine) and continuous fluoroscopic control. Ten minutes after the last alcohol injection the balloon is deflated and removed, ensuring that no alcohol backwash occurs into the left anterior descending artery. A final angiogram excludes LAD damage and verifies septal branch occlusion, and a final hemodynamic measurement is performed. The duration of postinterventional monitoring is controversially discussed. Since an artificial myocardial necrosis has been created, we suggest a monitoring duration of at least 48 hours (coronary or intensive care unit), with enzyme and ECG controls every 4 hours. Transvenous and transcutaneous pacing equipment should be readily available.

Absolute ethanol is not necessarily the only agent to induce the iatrogenic septal necrosis. Glue septal ablation using cyanoacrylate has been suggested to be a safe and effective approach to reduce septal thickness in patients with septal collateral vessels to the right coronary artery. The authors suggested that immediate glue polymerization prevents its transit through collateral vessels. Significant reductions in LVOT obstruction were observed, but long-term durability of this technique has not yet been demonstrated. Other cytotoxic agents that may be used are microcoils or contour emboli, and a small series with less favorable results reported on the use of radiofrequency energy [139, 140] applied either from the right ventricular septum or directly to the left ventricular septum.

### 4.2. Patient Selection for Septal Ablation

Criteria for patient selection largely follow those established for septal myectomy. Septal ablation may be considered an alternative to septal myectomy in [6, 8]:

1. patients with symptoms limiting daily activities (functional class > II, exercise-induced syncope) despite adequate medical treatment or if medical treatment is not tolerated;
2. patients with a substantial degree of outflow obstruction (pressure drop > 50–60 mm Hg with provocation by a Valsalva maneuver, bicycle stress, or postextrasystolic augmentation);
3. patients with a suitable left ventricular and coronary morphology, that is, those with a “classical,” subaortic obstruction produced by the protruding septum and the “SAM” of the mitral valve and one or more septal perforator arteries that go to this septal area.

Patients with coexisting, significant coronary artery disease in one vessel only may be treated percutaneously first; ablation should be delayed until documentation of a good long-term result of PCI. In cases with multiple (>1) vessel disease, we prefer a surgical approach. In atypical obstruction or midcavity obstruction, the decision must be individualized; ablation is possible [120, 130] but results are less favorable in this subgroup as compared to subaortic obstruction. At present, with respect to the very limited long-term experience with septal ablation and the favorable results of myectomy also in this age group, we are reluctant with ablation in the pediatric population with HOCM.

### 4.3. Current Results of Septal Ablation

As stated above, periprocedural mortality figures from experienced centers at present range between 0 and 2%. However, the rate of procedure-related pacemaker implantations still varies considerably, that is, between <5 and up to >20%. With respect to the morphologic and hemodynamic treatment result, across all reported series gradients usually are reduced by 80–90%, associated with an increase in exercise capacity, an improvement of diastolic LV function markers [132], and a reduction of left atrial size. A systematic review found a 30-day mortality of septal ablation around 1.5%, comparable to current survival rates after surgical myectomy. The most frequent complications of septal ablation were dissections of the LAD, cardiac tamponade, fatal bradyarrhythmias, ventricular fibrillation, cardiogenic shock, and pulmonary embolism. Agarwal and colleagues published a meta-analysis of twelve studies [122] comparing the short-term outcome of septal ablation and myectomy. They found no significant differences in short-term mortality (risk difference (RD): 0.01; 95% confidence interval (CI): −0.01 to 0.03).

Our own series now includes 603 patients (selected from a total of 1637 patients evaluated and treated in our HCM clinic). Out of these, 543 patients (90%) received an average dose of 2.4 ± 1.0 mL of ethanol. In 60 patients the intervention was aborted without ethanol injection, mostly for safety reasons/due to contrast echocardiographic findings. CK peak was 507 ± 246 U/L (normal value: <80). Transient AV conduction problems occurred in 245 patients (45%); permanent AV sequential pacing was required in 49 patients (9%). Peri-interventional mortality was 0.9% (5 deaths). After 3 months, self-reported exercise capacity improved in 493 patients (91%), with an average NYHA functional class improvement from 2.9 ± 0.4 to 1.6 ± 0.6; *P* < 0.01. Left ventricular outflow gradients were reduced from 62 ± 34 to 13 ± 21 mm Hg at rest and from 120 ± 36 to 41 ± 39 mm Hg with provocation (*P* < 0.0001). Septal thickness (from 20 ± 4 to 16 ± 4 mm; *P* < 0.01) and left atrial diameter (from 48 ± 7 to 45 ± 7 mm; *P* < 0.01) were also reduced. LV dilatation exceeding the individual normal value, or a global deterioration of systolic LV function, was not observed.

In addition to the comparable in-hospital mortality figures, the limited number of nonrandomized comparisons between septal ablation and (isolated) myectomy shows comparable clinical and hemodynamic results, with a slightly more pronounced improvement with respect to obstruction and exercise capacity following surgery and different surface ECG patterns (after septal ablation: RBBB; after myectomy: LBBB) after intervention [118, 121, 122, 124]. Whether these differences are clinically important or not is unknown. A difference that may be important is the fact that relief from obstruction is usually rapid after myectomy, whereas LV “unloading” after ablation may take several months.

The available publications on long-term effects of septal ablation showed that reduction of septal thickness and outflow gradient seems to continue over a 12-month period, presumably due to ongoing fibrosis and shrinking of the ethanol-induced septal lesion [110, 125–128, 131, 133, 134, 137]. Progressive LV dilatation was not observed; thus the remodeling process seems to remain limited to the region of intervention. Not only septal hypertrophy decreased as a consequence of the therapeutic infarction but also left ventricular posterior wall thickness due to relief of the pressure overload, which in turn indicates that the hypertrophic process in HOCM may not be completely independent of LV afterload. Overall 10-year survival was 90%; the event-free survival in NYHA class II or lower 76% figures again comparable to the reported postsurgical results [110, 131].

Concerns that septal scar induced by alcohol ablation might produce a new arrhythmogenic substrate have thus far not been validated. The long-term survival curves after surgical myectomy and septal ablation seem to be congruent [131, 134]. In one study that reported higher mortality and arrhythmogenic event [137] rates patients had received higher doses of ethanol than currently used. The question whether a successful septal alcohol ablation carries a prognostic benefit besides its symptomatic effect remains unanswered. Recent own data showed that survival in postablation HOCM patients was similar to that in an age-matched background population [131]. The number of risk factors, including the prevalence of nonsustained ventricular tachycardia, was reduced after ablation, and the incidence of sudden cardiac death was low. However, these findings must be confirmed by further investigations; currently we do not support a “prophylactic” intervention that addresses outflow gradients in asymptomatic patients. A meta-analysis comparing myectomy with septal ablation demonstrated absence of differences between the two procedures concerning the incidence of ventricular tachyarrhythmias.

## 5. Conclusion

Currently, for many patients with symptomatic HOCM, surgical myectomy and septal ablation can both be judged as reasonable options. Both procedures require extensive assessment and careful patient selection, should be performed by experienced operators in the context of a comprehensive program for HCM patients offering all other options (medical treatment, pacemaker and ICD implantation), result in a significant and long-standing clinical and hemodynamic benefit, and have a very acceptable safety profile. Consequently, the 2011 ACCF/AHA Guidelines for the Diagnosis and Management of HCM [8] advocate for septal ablation as a good alternative to surgery in those with significant comorbidity or advanced age and allow the procedure for those at lower surgical risk after a balanced discussion.

This discussion should refer to the individual anatomy, the likelihood of obtaining the desired result with a near-zero gradient, the comorbidities present, the available local expertise, and patient preference. Furthermore, both patient and operator should face the possibility that the ablation session might be ended without ethanol injection in case of lack of an appropriate septal target vessel. In our opinion ablation should be preferentially offered to older patients and to individuals with specific comorbidities and frailties in order to avoid the possible complications of open heart surgery. A preexisting left bundle-branch block increases the risk for pacemaker dependence after septal ablation to nearly 100%. Therefore, these patients preferably should undergo elective pacemaker implantation before ablation.

On the other hand, patients with extreme ventricular thickness (>30 mm) who more often also demonstrate marked myocardial fibrosis will probably have a less favorable outcome with alcohol ablation, and surgery remains a better choice. Surgery may also preferentially be offered in cases in which immediate relief from obstruction is an issue since the full effect of ablation may take several months. Furthermore, patients with concomitant multivessel coronary artery disease, mitral or aortic valve disease, or with anomalous papillary muscle insertion are candidates for operation.

Nearly twenty years after the first experimental cases, it thus appears reasonable to conclude that septal ablation and myectomy should no longer be seen as adversaries, but as partners in order to attain maximum patient benefit. A randomized trial comparing the two procedures has been and remains a major challenge for the future.
